# Mechanical C−C Bond Formation by Laser Driven Shock Wave

**DOI:** 10.1002/cphc.202000563

**Published:** 2020-08-26

**Authors:** Wakako Ishikawa, Shunichi Sato

**Affiliations:** ^1^ Institute of Multidisciplinary Research for Advanced Materials Tohoku University Aoba-ku Sendai 980-8577 Japan

**Keywords:** alkanes, C−C bond formation, gas chromatography, laser driven shock wave, mechanically induced reaction

## Abstract

Mechanically induced C−C bond formation was demonstrated by the laser driven shock wave generated in liquid normal alkanes at room temperature. Gas chromatography mass spectrometry analysis revealed the dehydrogenation condensation between two alkane molecules, for seven normal alkanes from pentane to undecane. Major products were identified to be linear and branched alkane molecules with double the number of carbons, and exactly coincided with the molecules predicted by supposing that a C−C bond was formed between two starting molecules. The production of the alkane molecules showed that the C−C bond formation occurred almost evenly at all the carbon positions. The dependence of the production on the laser pulse energy clearly indicated that the process was attributed to the shock wave. The C−C bond formation observed was not a conventional passive chemical reaction but an unprecedented active reaction.

## Graphical abstract

**Double up**: A mechanically induced chemical reaction was demonstrated by laser‐driven shock wave pressure in a liquid. Reduction of the molecular distance between alkane molecules by the laser shock wave gave rise to the formation of a C−C bond, creating an alkane molecule with double the number of carbon atoms. All combinations between carbon atoms for each alkane molecule were verified.

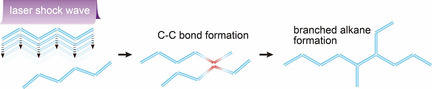

## 1 Introduction

When applying ultrahigh pressure on materials, the interatomic distances and the arrangement of atoms and molecules are remarkably changed. At 100 GPa, the intermolecular distances of materials were reduced by half compared with those at atmospheric pressure.^1^, ^2^ As a result, unprecedent physical and chemical phenomena were expected to occur. This feature has stimulated researches such as extraterrestrial material,^3^ structural phase transition,^4^, ^5^, ^6^ insulator‐metal transition,^7^ metallic hydrogen,^8^, ^9^ high‐temperature super conductor^10^, ^11^, ^12^ and dissociation/polymerization of molecules.^13^, ^14^, ^15^, ^16^ Ultrahigh pressure produced by diamond anvil cells^17^ is static and isotropic, whereas laser driven shock wave generates dynamic ultrahigh pressure^18^, ^19^, ^20^ up to 1 TPa^21^ compressing objects in one direction. Highly intense light field enough to generate such an ultrahigh pressure has been applied to atoms and molecules in vacuum. As a result, many physical and chemical phenomena such as high‐order harmonic generation,^22^ attosecond pulse generation,^23^ photoionization/dissociation^24^, ^25^ and Coulomb explosion,^26^ which produced radicals and fragment ions, have been investigated. However, the radicals and ions can hardly encounter to react each other in vacuum. By contrast, in liquid or solid, the radicals, ions, atoms and molecules would easily encounter to create a new molecule or species.^27^, ^28^ In an analogous way, when one directional and temporal compression force by laser shock wave is applied to molecules in liquid, it is expected that a chemical bond between molecules is created non‐thermally and mechanically by drastically reducing the intermolecular distance without significant deformation of molecular structure. Recently, a C−C bond formation was suggested for femtosecond laser irradiation of CO_2_ gas saturated water.^29^

In this research, we report that many kinds of alkane molecules with a larger number of carbons were synthesized through dehydrogenation condensation of two normal alkane molecules by applying laser driven shock wave even though alkanes are well known to be non‐polar and chemically stable. The number of carbon atoms of the major products was just twice that of starting molecule. For example, the irradiation of liquid hexane (C6) produced dodecane (C12) and its structural isomers such as 5‐methyl‐undecane and 4‐ethyl‐decane, which exactly coincided with those predicted by supposing that a C−C bond was formed between two hexane molecules. Other molecules with a smaller number of carbons were explained in the same way. The dependence of the production of the alkane molecules on the laser pulse energy indicated that the process was attributed not to multi‐photon absorption but to shock wave. This method can be referred to as mechanically induced chemical reaction since activation energy needed for reaction was provided in the form of mechanical energy. Although conventional chemical reactions strongly depended on thermal energy and occurred in a temporally random fashion, this method offered a tool to combine molecules at a controlled moment at room temperature.

## 2 Results and Discussion

Firstly, we will present the experimental results for normal hexane (C_6_H_14_) and show that major products were dodecane and its structural isomers (C_12_H_26_) indicating that two hexane molecules were combined releasing an H_2_ molecule, namely, dehydrogenation condensation. The synthesis obeyed a certain rule that the dodecane and its isomers (C12) identified were formed by the combination of carbons of each hexane molecule. Other alkane molecules with a smaller number of carbons (C_*n*_H_2*n*+2_; *n*<12) also obeyed a similar rule. Secondly, it will be shown that this phenomenon observed for hexane was universally found for other normal alkanes (*n*=5, 7, 8, 9, 10,11).

**Figure 1:**
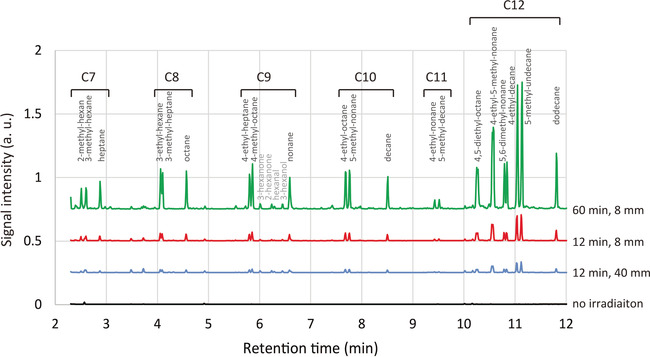
Gas chromatograms for normal hexane. Black line (bottom) is for the sample without laser irradiation. Other lines are for the samples irradiated by femtosecond laser pulses with an energy of 0.3 mJ and a repetition rate of 1000 Hz. The irradiation time was 12 min and 60 min for the blue and red lines, and the green line, respectively. Lenses with focal lengths of 40 mm and 8 mm were used for the blue line, and the red and green lines, respectively.

Figure 1 shows the chromatograms obtained for the normal hexane irradiated by focused femtosecond laser pulses. The pulse energy was 0.3 mJ and the repetition rate was 1000 Hz. The black line on the bottom was for the sample as purchased and not irradiated by laser pulses. The small peaks were originated from the impurities included in the normal hexane. When liquid hexane was irradiated for 12 min by using a lens of 40 mm focal length, many peaks appeared as shown in the blue line suggesting the production of molecules by laser irradiation. These peaks became higher when exchanged the focusing lens to another one with the shorter focal length of 8 mm as shown in the red line indicating the process was dependent on the laser intensity at the focus. The laser energy dependence will be discussed later in more detail. The green line shows the result when the irradiation time was extended to 60 min. The peak height increased about fivefold, indicating that the production was proportional to the irradiation time. Note that the experimental condition using the lens of 40 mm focal length as shown in the blue line was almost the same with that employed in the literatures,^30^, ^31^ which reported the formation of polyyne. The formation was attributed to the photodissociation of starting molecules and the recombination of the resultant radicals and fragment ions.^32^

In Figure 1, the name of identified molecule was shown above each peak of the chromatogram. In addition, the peaks were grouped by the number of carbons *n* as represented by C*n*. Briefly, the stronger peaks were distributed in the region with long retention time. These peaks were grouped as C12 and identified to be dodecane and its structural isomers, 5‐methyl‐undecane, 4‐ethyl‐decane, 5,6‐dimethyl‐decane, 4‐ethyl‐5‐methyl‐nonane and 4,5‐diethyl‐octane. The last three molecules showed a double peak in the chromatogram due to diastereomeric property. It should be noted that the number of carbons of these molecules is just double that of the starting molecule, hexane.

It is known that there are 355 structural isomers of dodecane, whereas only six isomers including dodecane were found in the experiment. If the molecular formation was accomplished by the recombination of radical, fragment ion, atom and molecule generated by photoionization and photodissociation of hexane in a similar way to polyyne formation,^32^ other isomers could be formed. Here we consider the combination between carbon atoms of each hexane molecule supposing that the C12 molecules found in the experiment were formed by binding two hexane molecules. To distinguish the position of carbon atom in a hexane molecule, each carbon is sequentially numbered from the edge. If a carbon at the edge (carbon 1) binds another carbon at the edge of each hexane, a dodecane molecule will be obtained accompanying the production of a hydrogen molecule. For the combination of carbon 1 and carbon 2, 5‐methyl‐undecane is obtained. 4‐ethyl‐5‐methyl‐nonane is for the combination of carbon 2 and carbon 3. Considering a symmetry of hexane molecule, six molecules in total can be obtained as summarized in Table 1. Surprisingly, the six molecules listed in Table 1 completely matched with those found in the experiment. This finding reminds us that there should be a formation rule to produce alkanes in our experimental condition.

**Table 1:** C12 molecules predicted by supposing that a carbon binds another carbon of each hexane molecule. Due to the symmetry of a hexane molecule, six molecules were deduced.

Carbon position	1	2	3
1	dodecane 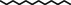	5‐methyl‐undecane 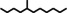	4‐ethyl‐decane 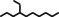
2		5,6‐dimethyl‐decane 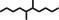	4‐ethyl‐5‐methyl‐nonane 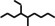
3			4,5‐diethyl‐octane 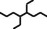

**Figure 2:**
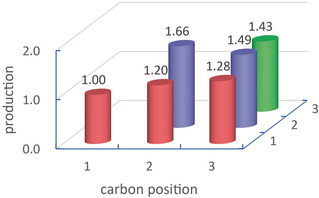
Relative production of the C12 molecules taking into account the number of combinations between the carbon positions of two hexane molecules for forming the molecule.

Next, the production volume of each C12 molecule was considered. The production of molecule was estimated from the area of corresponding peak of chromatogram. Since the peak heights in Figure 1 were seemingly random, the production of the C12 molecules seemed to be irregular. However, the production turned out to be rather regular after taking account of the number of combinations between carbons of each hexane molecule for forming each C12 molecule. For example, there are four combinations to form dodecane (carbon 1and carbon 1, carbon 1 and carbon 6, carbon 6 and carbon 1, and carbon 6 and carbon 6). For 5,6‐dimethyl‐decane and 4,5‐diethyl‐octane, the number of combinations is also four, whereas the combination number was eight for other C12 molecules such as 5‐methyl‐undecane, 4‐ethyl‐decane and 4‐ethyl‐5‐methyl‐nonane. Dividing the production simply estimated from the peak area by the number of combinations, the relative productions for the C12 molecules were obtained as shown in Figure 2. Here, the value for the carbon positions 1 and 1 was normalized to 1. The difference of production between the molecules was not as large as it seemed in Figure 1. Looking at the distribution carefully, it was noticed that the productions corresponding to the carbon position 1 were close to 1 but others were larger than 1.4. This difference can be attributed to the fact that the C−H bond dissociation energy of alkane depends on the carbon position. It is known that the C−H bond dissociation energy for carbon position 1, at which the carbon binds three hydrogens (–CH_3_) at the edge of a linear carbon chain, is higher than that for other carbon positions, at which the carbon binds two hydrogens (–CH_2_) inside the chain.^33^, ^34^ This means that the C−H bond at the carbon position 1 was more stable and the efficiency of C−C bond formation between hexane molecules was lower at the edge of a carbon chain.

**Figure 3:**
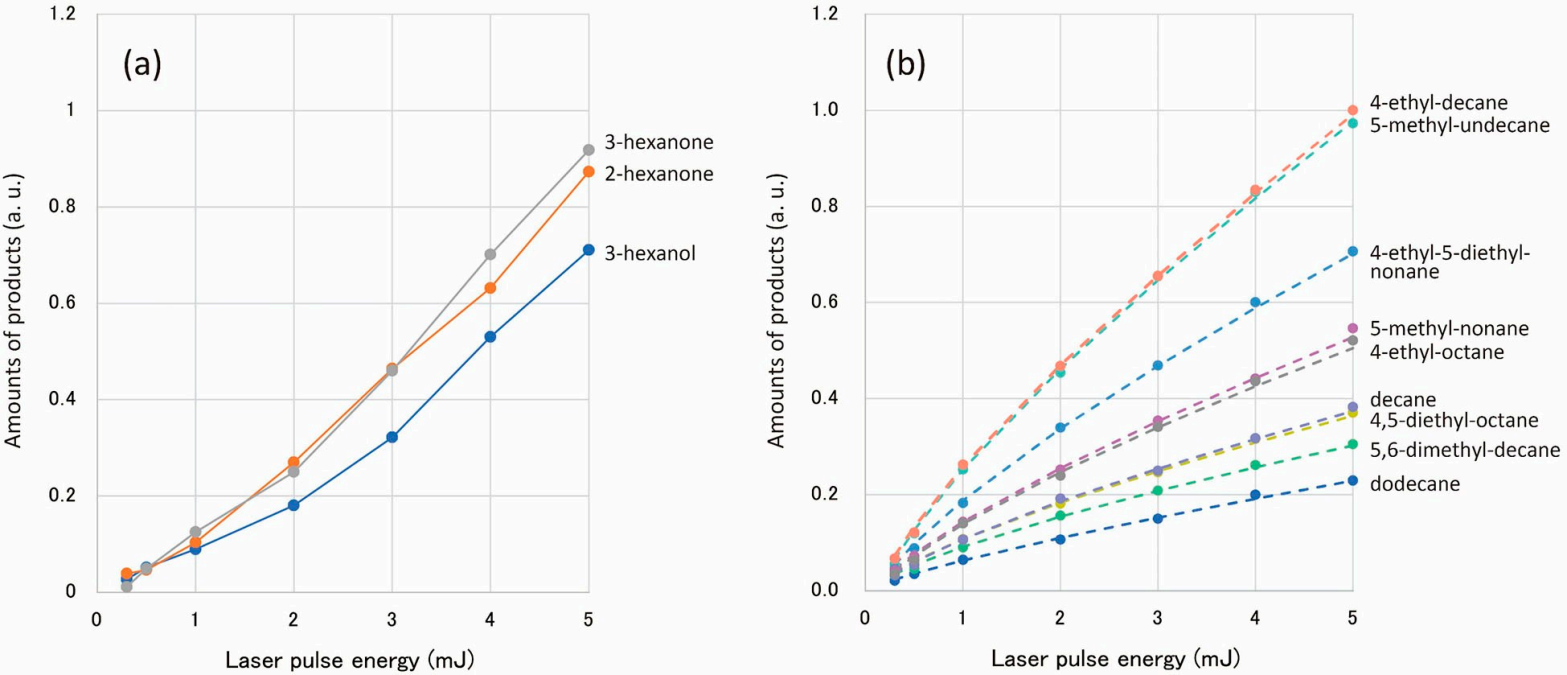
Amounts of products produced by the laser irradiation of liquid hexane as a function of the laser pulse energy for (a) oxygen‐containing organic compounds and (b) alkanes. (C10 and C12). The solid lines in (a) were just connecting the data points (closed circles). The dashed lines in (b) represented the fitting curves to the shifted power law.

Now we will see the dependence of the production on the pulse energy to investigate the formation mechanism. As seen in Figure 1, in addition to the peaks ascribed to alkanes, several oxygen‐containing molecules were identified near the retention time around 6 min. Figure 3 shows the amounts of products as a function of laser pulse energy for (a) the oxygen containing molecules and (b) the alkanes. The focusing lens of 8 mm focal length was used. The repetition rate and irradiation time were 10 Hz and 30 min, respectively. It is noted that the amounts of products are proportional to the signal intensity of corresponding chromatogram peak, whereas the comparison of the productions between different molecules can be performed by the peak area of the chromatogram. Although the production increased with increasing the pulse energy in both cases, the variations of slope were obviously different. In Figure 3 (a), the curves were concave up and the slope increased with the increase of the pulse energy like a non‐linear optical phenomenon such as multi‐photon absorption. In contrast, the curves in Figure 3 (b) were concave down. All the curves in Figure 3 (b) were remarkably similar to each other and approximated by the shifted power law expressed by *I*(*E*)=*C*(*E*−*E_th_*)^*a*^, where *E* is the pulse energy, *I*(*E*) is the amounts of products, *C* and *E*_th_ are constants and α is a variable. The curve fitting was performed for each molecule and represented by the dashed line in Figure 3 (b). The averaged best fits of α and *E*_th_ for nine alkanes were 0.76 and 0.10, respectively.

Suppose that the formation of the alkanes was attributed to laser shock wave. When a focal spot of a femtosecond laser beam is a point source of laser shock wave, the pressure of shock wave may be inversely proportional to the square of the distance from the source like acoustic wave. If the starting molecules, normal hexanes, can bind each other to form a C12 molecule in a region where the pressure of shock wave is above a threshold, the volume of reaction will be proportional to the cube of the distance. Considering that the laser shock wave pressure was proportional to the square root of the laser pulse energy,^35^ the production of C12 molecules will be proportional to three‐fourths (0.75) power of the pulse energy. This estimation was in excellent agreement with the experimental results in Figure 3 (b). Hence, the production of C12 alkanes observed in this experiment can be attributed to shock wave pressure. The threshold pulse energy deduced from the curve fitting also supported the generation and contribution of the shock wave. In contrast, for the oxygen‐containing molecules shown in Figure 3 (a), the different curve behaviour from Figure 3 (b) suggested that the formation mechanism differed from that for the C12 alkanes. In this case, oxygen atom could be supplied from dissolved molecular oxygen in liquid hexane. Because the number of oxygen atoms in those molecules was only one, molecular oxygen bond should be broken to incorporate an oxygen atom into a hexane molecule. The fact that the oxygen containing molecules were observed only for C6 compounds suggested that the formation mechanism differed from the photodissociation of hexane molecule, which was essential in the formation of polyyne.^32^

We will consider other alkanes containing a smaller number of carbon atoms, C7 to C11 alkanes. As seen in Figure 1, there were three kinds of alkanes for each C7 to C11 and their productions were low compared to C12. It should be noted that we can recognize regularity in their chemical structure, a normal alkane, a branched alkane with a methyl group and another branched alkane with an ethyl group, except for C11, probably due to quite low production of undecane. Interestingly, the combination rule found for the formation of C12 could be applied to C7 to C11, supposing that a part of hexane molecule is dropped out at the C−C bond formation. For example, C10 alkanes (decane, 5‐methyl‐nonane and 4‐ethyl‐octane) can be obtained by the combination of a hexane molecule with a second hexane molecule of which an ethyl group is dropped out. Decane corresponds to the combination of a hexane at the carbon position 1 with a second hexane at the carbon position 3 missing an ethyl group and keeping a butyl group. 5‐methyl‐nonane and 4‐ethyl‐octane are for the combination of hexanes at the carbon positions 2 and 3, respectively. For C11 to C7, the carbon positions of the second hexane molecule are 2 to 6 missing 1 to 5 carbons and corresponding hydrogens. In summary, C12 alkanes were formed retaining all the carbons of two hexane molecules, whereas alkanes from C7 to C11 lost carbons and related hydrogens at the occasion of C−C bond formation. In other words, C7 to C11 alkanes were formed by C−C bond formation between two hexane molecules in the similar way to C12 alkane formation but by dropping out a part of a hexane molecule accompanying C−C bond dissociation at the same time.

A resultant question is what the residuals resulted from the formation of C7 to C11 are. The possible candidates are C1 to C5 hydrocarbon molecules. Although the chromatogram in the shorter retention time region was not shown in Figure 1, C5 molecules such as pentane and pentene were found. The existence of alkanes and alkenes with a smaller number of carbons will be clearly shown for other alkanes with a larger number of carbons such as decane and undecane in the following.

**Figure 4:**
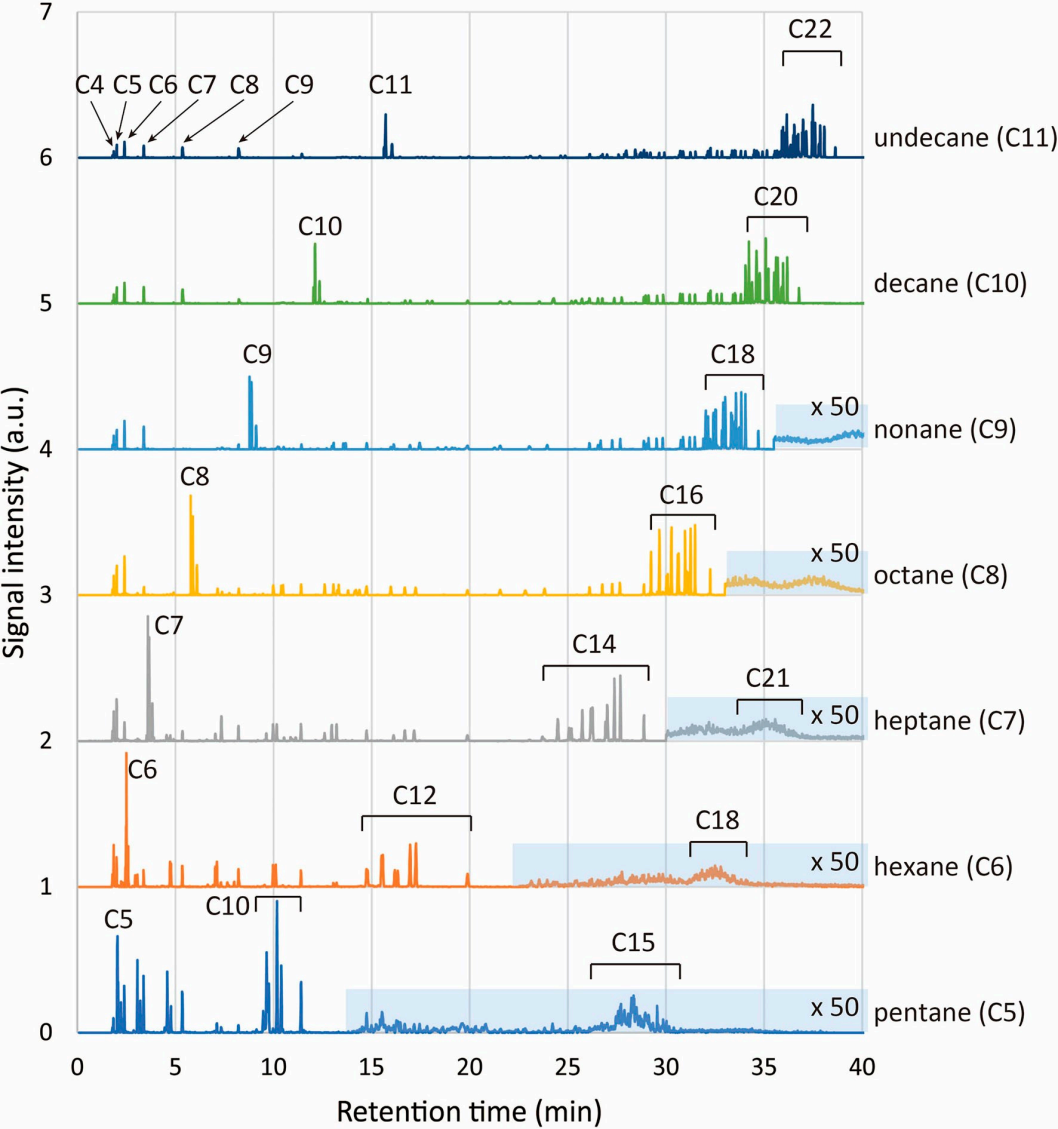
Chromatograms obtained for the femtosecond laser irradiation of liquid normal alkanes from pentane to undecane. Major products were indicated by their carbon numbers as C*n*. In the region filled in light blue, the signal intensity was multiplied by 50.

Laser irradiation of alkanes for pentane (C5) to undecane (C11) was performed to verify the consideration mentioned above. The repetition rate was 200 Hz and the irradiation time was 60 min. The laser pulses of 5.9 mJ was focused by a lens with the focal length of 8 mm. The signal to noise ratio of chromatogram was improved and the analysis of the peaks with small production became possible. Figure 4 shows chromatograms obtained for pentane to undecane from the bottom to the top. For each chromatogram, the data of the starting alkane was subtracted from the data of laser irradiated sample. Accordingly, only the peaks produced by laser irradiation were shown.

**Figure 5:**
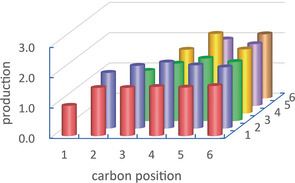
Relative production of C22 molecules taking into account the number of combinations between the carbon positions of two undecane molecules for forming the molecule.

Recognizable peaks in Figure 4 were identified to be alkanes and alkenes except for a small number of peaks corresponding to oxygen containing molecules. In the following, we will focus on the chromatogram obtained for undecane because typical features common to all the chromatograms appeared in it. Higher peaks were located in the longest retention time region as seen for hexane in Figure 1. As predicted by the combination rule between two undecane molecules, these peaks were identified to be 21 alkanes (C22) including docosane and its structural isomers as summarized in Table 2. These alkanes completely coincided with the molecules predicted by the combination rule for undecane. The production of each alkane was depicted in Figure 5. Here the production was normalized to the production of docosane, which corresponded to the combination of carbon positions 1 and 1 of each undecane. Minimum production was observed for docosane, whose C−H bond dissociation energy was extrapolated to be highest from the analogy of hexane and heptane.^33^, ^34^ The productions of alkanes that combined at the carbon position 1 of one undecane were low compared to other alkanes. As a whole, the production increased with shifting the carbon position of C−C bond formation from the edge to the center of undecane with a little undulation probably due to small differences of C−H and C−C bond dissociation energies between carbon positions.^36^ These features observed for undecane resembled those for hexane as shown in Figure 2. However, the productions for undecane were relatively higher than those for hexane. This margin was unable to ascribe to bond dissociation energy. This problem will be discussed later again.

**Table 2:** List of molecules for the femtosecond laser irradiation of liquid undecane. C22 alkanes were predicted by the combination of carbons of each undecane molecule. For C12 to C21, it was supposed that a part of undecane was dropped out at the C−C bond formation. The residuals of the undecane were assumed to form alkanes and alkenes for C4 to C10. Asterisk indicates the molecule was not detected.

Carbon number	Molecules
C22	docosane, 10‐methyl‐henicosane, 9‐ethyl‐eicosane, 10,11‐dimethyl‐eicosan, 8‐propyl‐nonadecane, 7‐butyl‐octadecane, 6‐pentyl‐heptadecane, 9‐ethyl‐10methyl‐nonadecane, 8‐propyl‐9‐methyl‐octadecane, 9,10‐diethyl‐octadecane, 7‐butyl‐8‐methyl‐octadecane, 6‐pentyl‐7‐methyl‐hexadecane, 9‐ethyl‐8‐propyl‐heptadecane, 7‐butyl‐8‐ethyl‐hexadecance, 7‐ethyl‐6‐pentyl‐pentadecane, 8,9‐dipropyl‐hexadecane, 7‐butyl‐8‐propyl‐pentadecane, 6‐pentyl‐7‐propyl‐tetradecane, 7,8‐dibutyl‐tetradecane, 7‐butyl‐6‐pentyl‐tridecane, 6,7‐dipentyl‐dodecane
C21	heneicosane, *10‐methyl‐eicosane, *9‐ethyl‐nonadecane, *8‐propyl‐octadecane, *7‐butyl‐heptadecane, *6‐pentyl‐hexadecane
C20	eicosane, *10‐methyl‐nonadecane, *9‐ethyl‐octadecane, 8‐propyl‐heptadecane, 7‐butyl‐hexadecane, 6‐pentyl‐pentadecane
C19	nonadecane, 9‐methyl‐octadecane, 9‐ethyl‐heptadecane, 8‐propyl‐hexadecane, 7‐butyl‐pentadecane, 6‐pentyl‐tetradecane
C18	octadecane, 8‐methyl‐heptadecane, 8‐ethyl‐hexadecane, 8‐propyl‐pentadecane, 7‐butyl‐tetradecane, 6‐pentyl‐tridecane
C17	heptadecane, 7‐methyl‐hexadecane, 7‐ethyl‐pentadecane, 7‐propyl‐tetradecane, 7‐butyl‐tridecane, 6‐pentyl‐dodecane
C16	hexadecane, 6‐methyl‐pentadecane, 6‐ethyl‐tetradecane, 6‐propyl‐tirdecane, 6‐butyl‐dodecane, 6‐pentyl‐undecane
C15	pentadecane, 5‐methyl‐tetradecane, 5‐ethyl‐tridecane, 5‐propyl‐dodecane, 5‐butyl‐undecane, 5‐pentyl‐decane
C14	tetradecane, 4‐methyl‐tridecane, 4‐ethyl‐dodecane, 4‐propyl‐undecane, 4‐butyl‐decane, 4‐pentyl‐nonane
C13	tridecane, 3‐methyl‐dodecane, 3‐ethyl‐undecane, 3‐propyl‐decane, 3‐butyl‐nonane, 3‐pentyl‐octane
C12	*dodecane, *3‐methyl‐undecane, *2‐methyl‐decane, *4‐methyl‐decane, *5‐methyl‐decane, *6‐methyl‐decane
C11	(*E*)‐2‐undecene, (*Z*)‐2‐undecene
C10	*decane, *1‐decene
C9	nonane, 1‐nonene
C8	octane, 1‐octene
C7	heptane, 1‐heptene
C6	hexane, 1‐hexene
C5	pentane, 1‐pentene
C4	butane, 1‐butene

For C12 to C21, a lot of small peaks were observed at the shorter retention time region. The molecules predicted by the combination rule were listed in Table 2. Six alkanes were inferred for each carbon number by assuming that the carbons of one undecane was preserved but another one was broken into two at the C−C bond formation. The production was relatively low for C12, C20 and C21, which corresponded to the combination with the carbon at the edge or near the edge. In the experiment, 13 molecules in total were not observed for these carbon numbers as indicated by asterisk in Table 2. In contrast, the peak heights for C13 to C19 were relatively high and mostly the same each other as seen in Figure 4. Around the retention time of 16 min, two peaks identified to be (E)‐2‐undecene and (Z)‐2‐undecene were clearly seen. These alkenes (C_11_H_22_) have a double bond between the carbon positions 2 and 3. Because the chromatogram was obtained by subtracting the data of the liquid undecane as purchased, these molecules should be formed by the laser irradiation. Possible production route was directly induced photo‐dehydrogenation of undecane molecule or failure of C−C bond formation between undecane molecules. If so, 1‐undecene with a double bond between the carbon positions 1 and 2 would be another candidate as a product. Because the retention time of 1‐undecene considerably overlapped that of undecane, whose signal in the chromatogram was subtracted in Figure 4, it was difficult to show the evidence of 1‐undecene. However, 1‐alkenes for C4 to C9 such as 1‐butene and 1‐nonane were identified, even though the retention time of 1‐alkenes also overlapped those of normal alkanes with the same number of carbons. Note that decane and 1‐decene were not recognized because a significant amount of decane was included in liquid undecane as impurity. Thus, the existence of 1‐alkenes suggested that a part of undecane was dropped out at ultrahigh pressure induced C−C bond formation, resulting in the production of alkanes and alkenes with a smaller number of carbons, C4 to C10 at least. It should be noted that the boiling points of C1 to C3 alkanes and alkenes are around 230 K or lower. Accordingly, these C1 to C3 molecules were hardly detected by the GC‐MS even if those were produced in the sample.

In Figure 4, the chromatograms for normal alkanes, pentane (C5) to undecane (C11), were depicted. They resembled each other, especially for alkanes with a larger number of carbons, supporting that the production mechanism was the C−C bond formation by laser driven ultrahigh pressure as discussed above. All the recognizable peaks were identified to be alkanes and alkenes, which were predicted by the combination rule, excepted for some oxygen containing molecules. As seen in Figure 4, the highest peaks were identified to be alkanes with double carbon numbers (C2*n*: *n* is integer) for each starting alkane (C*n*). Two peaks corresponding to 2‐alkenes (C*n*) were clearly recognized. In addition, overlapped peaks of alkane and 1‐alkene were also observed at the same positions of each chromatogram. Furthermore, very small but a large number of peaks appeared at the retention time shorter than 35 min for the liquid hexane as shown as the second curve from the bottom in Figure 4. Because this retention time region corresponded to C18 alkanes as seen for liquid nonane (C9), these peaks can be attributed to secondary products (C18) by the combination of hexane (C6) and C12 alkanes primarily produced by the laser irradiation. According to the combination rule, hexane and six alkanes listed in Table 1 will produce 162 alkanes considering symmetries of the molecules. Thus, it is not easy to identify each peak but acceptable to ascribe those small peaks below 35 min in the chromatogram to secondarily produced C18 alkanes. Furthermore, a lot of much smaller peaks below the retention of 30 min were thought to be the secondary products with C9 to C11 alkanes. These secondary products were also recognized for other alkanes, pentane, heptane, octane and nonane.

Looking at the chromatograms in Figure 4 carefully, the peaks corresponding to C*n*+1 to C2*n*‐1 became higher with decreasing the carbon number of the starting alkane. This suggests that the breaking of carbon chain at the C−C bond formation was more difficult for longer chain alkanes. In other words, longer chain alkane might be resilient to mechanical force produced by the laser driven shock wave. This consideration implies that mechanical property of molecule may play an important role in the reaction. In addition, this resilient property of longer carbon chain would be related to the increase of production for undecane shown in Figure 5.

## 3 Conclusion

We demonstrated the formation of many alkanes and alkenes by femtosecond laser irradiation of liquid normal alkane. Identification of the molecules revealed an obvious formation rule for the alkanes and alkenes produced. For normal alkane with the carbon number of *n* (C*n*) as a starting material, identified C2*n* alkanes were exactly coincident with those predicted by supposing that a C−C bond was formed between two alkanes. For the molecules with a smaller number of carbons (C*n*+1 to C2*n*−1), the same formation rule was applicable except for the dropout of a fraction of alkane, which corresponded to the alkanes and alkenes with a smaller number of carbons than *n*. The dependence of the production of identified alkanes on the laser pulse energy indicated that the formation of the alkanes was attributed to the laser driven shock wave. Thus, it was concluded that a mechanically induced chemical reaction was essential for the observed results in the experiment.

## Experimental Section

Normal alkanes (FUJIFILM Wako Pure Chemical Corp.), pentane, hexane, heptane, octane, nonane, decane and undecane, were used for experiment as purchased without further purification. A 2 mL aliquot of each liquid alkane contained in a rectangular quartz cuvette with the pass length of 10 mm was irradiated by femtosecond laser pulses with the pulse energy up to 6 mJ, the pulse width of 100 fs, the repetition rate up to 1000 Hz, the beam diameter of 10 mm and the wavelength of 800 nm from a Ti:sapphire regenerative amplifier (Spitfire Pro, Spectra‐Physics). The laser pulses were focused near the center of the cuvette by a plano‐convex lens with the focal length of 40 mm or an aspheric lens with the focal length of 8 mm. The laser intensity at the focus was estimated to be 2.3×10^14^ W/cm^2^ for the aspheric lens supposing that the beam diameter at the focal point was 175 μm considering the spherical aberration at the interfaces between air, glass and liquid. The laser irradiated samples were analyzed by a gas chromatography‐mass spectrometry system (GC‐MS; Agilent 6890 N and 5975 C). The column was DB‐1701.

## Conflict of interest

The authors declare no conflict of interest.

## References

[1] P. F. McMillan , Chem. Soc. Rev. 2006, 35, 855–857. 17003892 10.1039/b610410j

[2] P. Loubeyre , R. LeToullec , D. Hausermann , M. Hanfland , R. J. Hemley , H. K. Mao , L. W. Finger , Nature 1996 383, 702–704.

[3] L. R. Benedetti , J. H. Nguyen , W. A. Caldwell , H. Liu , M. Kruger , R. Jeanloz , Science 1999, 5437, 100–102. 10.1126/science.286.5437.100 10506552

[4] F. McMillan , Nat. Mater. 2002,1, 19–25. 12618843 10.1038/nmat716

[5] M. Angel Rubio , A. Muñoz , R. J. Needs , Rev. Mod. Phys. 2003, 75, 863–912.

[6] S. Wong , T. B. Shiell , B. A. Cook , J. E. Bradby , D. R. McKenzie , D. G. McCulloch , Carbon 2019, 142, 473–481.

[7] H. .-K. Mao , X.-J. Chen , Y. Ding , B. Li , L. Wang , Rev. Mod. Phys. 2018, 90, 015007.

[8] E. Wigner , H. B. Huntington , J. Chem. Phys. 1935, 3, 764–770.

[9] J. McMinis , R. C. Clay, III , D. Lee , M. A. Morales , Phys. Rev. Lett. 2015, 114, 105305. 25815944 10.1103/PhysRevLett.114.105305

[10] A. P. Drozdov , M. I. Eremets , I. A. Troyan , V. Ksenofontov , S. I. Shylin , Nature 2015, 525, 73–76. 26280333 10.1038/nature14964

[11] M. Somayazulu , M. Ahart , A. K. Mishra , Z. M. Geballe , M. Baldini , Y. Meng , V. V. Struzhkin , R. J. Hemley , Phys. Rev. Lett. 2019, 122, 027001. 30720326 10.1103/PhysRevLett.122.027001

[12] A. P. Drozdov , P. P. Kong , V. S. Minkov , S. P. Besedin , M. A. Kuzovnikov , S. Mozaffari , L. Balicas , F. F. Balakirev , D. E. Graf , V. B. Prakapenka , E. Greenberg , D. A. Knyazev , M. Tkacz , M. I. Eremets , Nature 2019, 569, 528–531. 31118520 10.1038/s41586-019-1201-8

[13] K. Aoki , S. Usuba , M. Yoshida , Y. Kakudate , K. Tanaka , S. Fujiwara , J. Chem. Phys. 1988, 89, 529–534.

[14] A. F. Goncharov , M. Riad Manaa , J. M. Zaug , R. H. Gee , L. E. Fried , W. B. Montgomery , Phys. Rev. Lett. 2005, 94, 065505. 15783746 10.1103/PhysRevLett.94.065505

[15] K. Takemura , K. Sato , H. Fujihisa , M. Onoda , Nature 2003, 423, 971–974.12827197

[16] V. Schettino , R. Bini , Phys. Chem. Chem. Phys. 2003, 5, 1951–1965.

[17] E. F. O'Bannon III , Z. Jenei , H. Cynn , M. J. Lipp , J. R. Jeffries , Rev. Sci. Instrum. 2018, 89, 111501. 30501343 10.1063/1.5049720

[18] D. G. Hicks , T. R. Boehly , P. M. Celliers , J. H. Eggert , S. J. Moon , D. D. Meyerhofer , G. W. Collins , Phys. Rev. B 2009, 79, 014112.

[19] M. Guarguaglini , J.-A. Hernandez , T. Okuchi , P. Barroso , A. Benuzzi-Mounaix , M. Bethkenhagen , R. Bolis , E. Brambrink , M. French , Y. Fujimoto , R. Kodama , M. Koenig , F. Lefevre , K. Miyanishi , N. Ozaki , R. Redmer , T. Sano , Y. Umeda , T. Vinci , A. Ravasio , Sci. Rep. 2019, 9, 10155. 31300690 10.1038/s41598-019-46561-6 PMC6626017

[20] D. S. Moore , J. Opt. Soc. Am. B 2018, 35, B1–B15.

[21] A. Fernandez-Pañella , M. Millot , D. E. Fratanduono , M. P. Desjarlais , S. Hamel , M. C. Marshall , D. J. Erskine , P. A. Sterne , S. Haan , T. R. Boehly , G. W. Collins , J. H. Eggert , P. M. Celliers , Phys. Rev. Lett. 2019, 122, 255702. 31347873 10.1103/PhysRevLett.122.255702

[22] A.-T. Le , R. R. Lucchese , S. Tonzani , T. Morishita , C. D. Lin , Phys. Rev. A 2009, 80, 013401.

[23] F. Krausz , M. Ivanov , Rev. Mod. Phys. 2009, 81, 163–234.

[24] M. Castillejo , S. Couris , E. Koudoumas , M. Martín , Chem. Phys. Lett. 1999, 308, 373–380.

[25] M. F. Kling , C. Siedschlag , A. J. Verhoef , J. I. Khan , M. Schultze , T. Uphues , Y. Ni , M. Uiberacker , M. Drescher , F. Krausz , M. J. J. Vrakking , Science 2006, 312, 246–248. 16614216 10.1126/science.1126259

[26] J. H. Posthumus , Rep. Prog. Phys. 2004, 67, 623–665.

[27] A. Hu , J. Sanderson , A. A. Zaidi , C. Wang , T. Zhang , Y. Zhou , W. W. Duley , Carbon 2008, 46, 1823–1825.

[28] A. Ramadhan , M. Wesolowski , T. Wakabayashi , H. Shiromaru , T. Fujino , T. Kodama , W. Duley , J. Sanderson , Carbon 2017, 118, 680–685.

[29] T. Nishi , S. Sato , A. Ohshima , T. Nakamura , S. Sato , T. Morikawa , Jpn. J. Appl. Phys. 2020, 59, 057001.

[30] Y. Sato , T. Kodama , H. Shiromaru , J. H. Sanderson , T. Fujino , Y. Wada , T. Wakabayashi , Y. Achiba , Carbon 2010, 48, 1673–1676.

[31] A. A. Zaidi , A. Hu , M. J. Wesolowski , X. Fu , J. H. Sanderson , Y. Zhou , W. W. Duley , Carbon 2010, 48, 2517–2520.

[32] A. A. Zaidi , A. Hu , D. E. Henneke , W. W. Duley , Chem. Phys. Lett. 2019, 723, 151–154.

[33] J. M. Hudzik , J. W. Bozzelli , J. M. Simmie , J. Phys. Chem. A 2014, 118, 9364–9379. 25180943 10.1021/jp503587b

[34] J. Zheng , T. Yu , D. G. Truhlar , Phys. Chem. Chem. Phys. 2011, 13, 19318–19324. 21984114 10.1039/c1cp21829h

[35] E. N. Glezer , C. B. Schaffer , N. Nishimura , E. Mazur , Opt. Lett. 1997, 22, 1817–189. 18188376 10.1364/ol.22.001817

[36] J. Wu , H. Ning , L. Ma , W. Ren , Chem. Phys. Lett. 2018, 699, 139–145.

